# Stromal-derived factor-1α/CXCL12-CXCR4 chemotactic pathway promotes perineural invasion in pancreatic cancer

**DOI:** 10.18632/oncotarget.3069

**Published:** 2014-12-26

**Authors:** Qinhong Xu, Zheng Wang, Xin Chen, Wanxing Duan, Jianjun Lei, Liang Zong, Xuqi Li, Liang Sheng, Jiguang Ma, Liang Han, Wei Li, Lun Zhang, Kun Guo, Zhenhua Ma, Zheng Wu, Erxi Wu, Qingyong Ma

**Affiliations:** ^1^ Department of Hepatobiliary Surgery, First Affiliated Hospital of Medical College, Xi'an Jiaotong University, Xi'an 710061, Shaanxi, China; ^2^ Department of Oncology, First Affiliated Hospital of Medical College, Xi'an Jiaotong University, Xi'an 710061, Shaanxi, China; ^3^ Department of General Surgery, First Affiliated Hospital of Medical College, Xi'an Jiaotong University, Xi'an 710061, Shaanxi, China; ^4^ Department of Pharmaceutical Sciences, North Dakota State University, Fargo, ND 58105, USA

**Keywords:** CXCL12/CXCR4 axis, Perineural Invasion, Tumor Microenvironment, Pancreatic Cancer

## Abstract

Perineural invasion (PNI) is considered as an alternative route for the metastatic spread of pancreatic cancer cells; however, the molecular changes leading to PNI are still poorly understood. In this study, we show that the CXCL12/CXCR4 axis plays a pivotal role in the neurotropism of pancreatic cancer cells to local peripheral nerves. Immunohistochemical staining results revealed that CXCR4 elevation correlated with PNI in 78 pancreatic cancer samples. Both *in vitro* and *in vivo* PNI models were applied to investigate the function of the CXCL12/CXCR4 signaling in PNI progression and pathogenesis. The results showed that the activation of the CXCL12/CXCR4 axis significantly increased pancreatic cancer cells invasion and promoted the outgrowth of the dorsal root ganglia. CXCL12 derived from the peripheral nerves stimulated the invasion and chemotactic migration of CXCR4-positive cancer cells in a paracrine manner, eventually leading to PNI. *In vivo* analyses revealed that the abrogation of the activated signaling inhibited tumor growth and invasion of the sciatic nerve toward the spinal cord. These data indicate that the CXCL12/CXCR4 axis may be a novel therapeutic target to prevent the perineural dissemination of pancreatic cancer.

## INTRODUCTION

Pancreatic cancer (PCa) is now the fourth most lethal cancer with a median survival time of less than 6 months and a 5-year survival rate of < 6% [1]. Due to a lack of early symptoms, most PCa cases are advanced at the time of diagnosis, and only approximately 20% of patients are considered eligible for resection [2, 3]. The hallmarks of this type cancer include rapid progression, short survival durations, and resistance to therapy. The poor prognosis of PCa is related to its local recurrence, lymph node and liver metastases, peritoneal dissemination and perineural invasion (PNI) [4, 5]. The vital role of PNI for the recurrence and metastasis in PCa has been widely recognized, but the underlying mechanisms still require elucidation.

PNI is considered an independent prognostic factor which is characteristic of PCa. It is estimated that up to 90% of patients have intra-pancreatic nerve infiltration by tumor cells, and 69% patients have involvement of the extra-pancreatic nerve terminations [6]. Our previous study reported a PNI rate of 86.9% (53/61) in PCa patients [7]. It is reported that the incidence of PNI reaches nearly 100% if enough surgical specimen sections are evaluated [3, 8]. The presence of tumor cells in the perineurium space of the local pancreatic peripheral nerves might be associated with the abdominal pain sensation and the higher risk of local recurrence after tumor resection. The tumor cells initially infiltrate into the surrounding nerves and then migrate along the nerves. Thus, PNI provides an alternative route for metastatic spread [3, 9]. There are two prominent theories regarding PNI: one is the “path of low resistance”, which mainly focuses on anatomy [10]; while the other involves reciprocal signaling interactions and emphasizes the molecular mechanism between nerves and the invading tumor cells. More recently, studies have found PNI to be closely related to certain factors, including nerve growth factor (NGF), brain-derived neurotrophic factor (BDNF), neurotrophin-3 (NT-3), neural cell adhesion molecules (NCAM), glial cell line-derived neurotrophic factor (GDNF) and matrix metalloproteinases (MMPs) [4, 11]. A recent study reported that the chemokine receptor CX3CR1 is involved in neural tropism and the malignant behavior of PCa [12].

Chemokines and their receptors have been implicated in tumor growth and tumor cell invasion of surrounding organs. The CXCL12/CXCR4 axis is one of the most widely studied chemokine signaling pathways. CXCR4 selectively binds to CXC chemokine stromal cell-derived factor 1α (SDF-1α or CXCL12), and CXCL12/CXCR4 signaling is regarded as a candidate factor involved in the tumor-stromal interactions. CXCR4-positive tumor cells migrate toward distant organs in response to a CXCL12 gradient [13]. This axis has been found to play important roles in the proliferation, angiogenesis, epithelial–mesenchymal transition, metastasis and invasion of several cancers, including PCa [14-20]. CXCL12 and CXCR4 have been detected in the central and peripheral nervous systems, and the neurons as well as Schwann cells are the major producers of the ligand [21, 22]. The chemokine CXCL12 and its receptor CXCR4 control the migration of neurons and microglial cells in the central nervous system [23]. Whether the CXCL12 secreted by peripheral nerves mediates the chemotactic migration of PCa cells is an intriguing question.

The continuous expression of CXCL12 in neural cells and the high frequency of PNI in pancreatic adenocarcinoma prompted us to test the hypothesis that the CXCL12/CXCR4 signaling pathway is involved in PNI in PCa. An optimum dose gradient of CXCL12 conducted by the neurons and Schwann cells induces the chemotactic migration of CXCR4-bearing tumor cells toward the peripheral nerves, and simultaneously increases PCa cells metastasis and invasion, thereby establishing PNI.

In the present study, an *in vitro* model generated by co-culturing newborn rat dorsal root ganglia (DRG) and PCa cells as well as an *in vivo* PNI model were applied to investigate the function of CXCL12/CXCR4 signaling in PNI progression and pathogenesis. We showed that the peripheral nerve-derived CXCL12 stimulated the invasion and chemotactic migration of CXCR4-positive cancer cells in a paracrine manner, eventually leading to PNI. These data indicate that the CXCL12/CXCR4 axis is involved in PNI, and the inhibition of the signaling pathway may be a promising new therapeutic target for PNI and tumor recurrence in PCa.

## RESULTS

### Expression and clinical significance of CXCR4 and CXCL12 in PCa

**Fig. 1:**
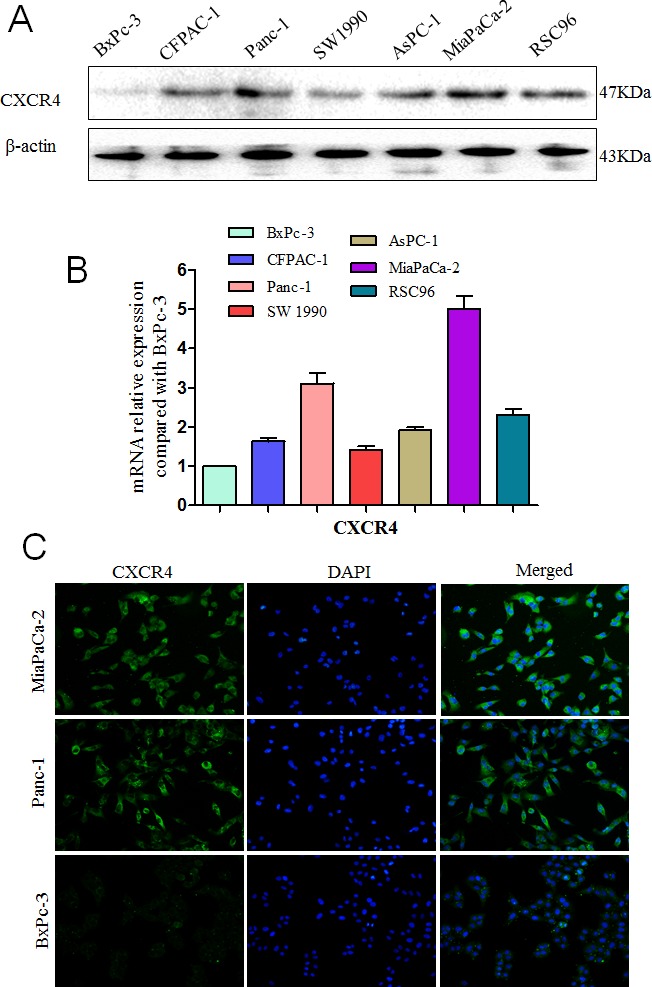
Expression levels of CXCR4 and CXCL12 in pancreatic cancers The expression level of the CXCR4 protein (A) and mRNA (B) in six PCa cell lines (BxPC-3, CFPAC-1, Panc-1, SW1990, AsPC-1, MiaPaCa-2) and Schwann cell RSC96; (C) MiaPaCa-2, Panc-1 cells and BxPc-3 cells were labeled with a fluorescence-conjugated CXCR4-specific antibody (green). Nuclei were stained with DAPI (blue; 200× magnification).

We first examined the expression of CXCR4 and CXCL12 in PCa cells and found high CXCR4 levels in all six PCa cell lines. The expression of the CXCR4 gene is 1.63-fold (CFPAC-1), 3.11-fold (Panc-1), 1.42-fold (SW1990), 1.92-fold (AsPC-1), 5.01-fold (MiaPaCa-2) and 2.32-fold (RSC96) higher than that in BxPc-3 cells (Fig. 1A and B). CXCL12 expression is rarely detected by ELISA (Fig. 4C), and is not detected by qRT-PCR or western blot assays. Among the six cell lines, MiaPaCa-2 had the highest CXCR4 expression. Immunofluorescence showed that CXCR4 is localized to the cytoplasm and the membrane of the MiaPaCa-2 and Panc-1 cells; and BxPc-3 cells are used as a control (Fig. 1C).

**Fig. 2:**
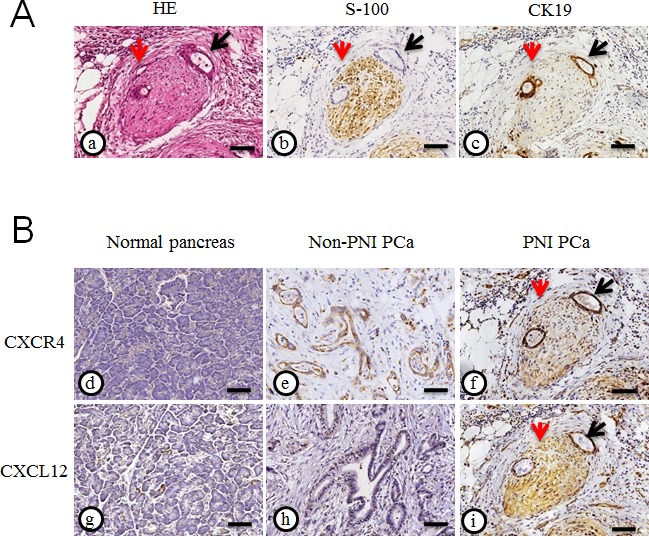
Expression of CXCR4 and CXCL12 in pancreatic cancer tissues (A) HE staining (a), immunohistochemical staining of S100 (b) served as a nerve tissue marker and CK19 (c) served as a cancer cell marker in PCa tissues with PNI; (B) The representative immunohistochemical staining for CXCR4 and CXCL12 in the normal pancreas (d and g) and the resected PCa specimens accompanied without PNI (e and h) or with PNI (f and i) (200× magnification); the peripheral nerve (red arrow) was infiltrated by PCa cells (black arrow). Scale bar, 100μm.

In our previous study, we found that the overall survival patients with positive CXCR4 expression is significantly lower than that of patients negative for CXCR4. CXCR4 overexpression correlated with an advanced cancer stage and metastasis. To explore the novel role of the CXCL12/CXCR4 axis in PNI, we evaluated the representative immunohistochemical staining properties of CXCR4 and CXCL12 in the resected PCa specimens accompanied by PNI, where the staining of S100 served as a nerve tissue marker and CK19 served as a cancer cell marker (Fig. 2A). As shown in Fig. 2B, a majority of cancer cells and nerve tissues showed distinct immunostaining of CXCR4 and CXCL12 localized to the cytoplasm. The expression of PNI PCa tissues is significantly increased compared with that of the non-PNI PCa tissues.

Next, we determined the correlation between CXCR4/CXCL12 expression and PNI in histological sections of PCa samples. Out of a total of 78 resected PCa samples, 62 (79.5%) are positive, and 16 (20.5%) are negative for CXCR4 staining. The incidence of PNI is as high as 67.9%. The χ^2^ analysis revealed that histologic markers of aggressive disease, including positive lymph node metastasis (P=0.045), TNM stage (P=0.015), vascular invasion (P=0.049), and especially PNI (P=0.0001) are significantly associated with CXCR4 overexpression. The expression level of CXCL12 is higher in the groups with lymph node metastasis, vascular invasion and PNI, although there is no significant difference. CXCL12 expression in PCa do not correlate with any clinicopathologic features. (Table 1).

**Table 1:** The relationship between expression of CXCR4/CXCL12 and clinicopathological features in 78 cases of PCa

		CXCR4 n (%)			CXCL12 n (%)		
	All Cases	Negative n=16 (20.5%)	Positive n=62 (79.5%)	*P* Value^∆^	Negative n=24 (20.5%)	Positive n=54 (79.5%)	*P* Value^∆^
Sex				0.626			0.698
Male	48	9 (18.8)	39 (81.2)		14 (29.2)	34 (70.8)	
Female	30	7 (23.3)	23 (76.7)		10 (33.3)	20 (66.7)	
Age (Years)				0.146			0.241
≤57^†^	41	11 (26.8)	30 (73.2)		15 (36.6)	26 (63.4)	
>57	37	5 (13.5)	32 (86.5)		9 (24.3)	28 (75.7)	
Histological grade				0.691			0.196
Well	23	5 (21.7)	18 (78.3)		6 (26.1)	17 (73.9)	
Moderate	36	6 (16.7)	30 (83.3)		9 (25.0)	27 (75.0)	
Poor	19	5 (26.3)	14 (73.7)		9 (47.4)	10 (52.6)	
Lymph node metastasis				0.045^*^			0.639
Negative	49	14 (28.6)	35 (71.4)		16 (32.7)	33 (67.3)	
Positive	29	2 (6.9)	27 (93.1)		8 (27.6)	21 (72.4)	
TNM stage (AJCC)				0.037^*^			0.868
I	23	9 (39.1)	14 (60.9)		8 (34.8)	15 (65.2)	
II	35	6 (17.1)	29 (82.9)		10 (28.6)	25 (71.4)	
III	9	1 (11.1)	8 (88.9)		2 (22.2)	7 (77.8)	
IV	11	0 (0)	11 (100.0)		4 (36.4)	7 (63.6)	
Vascular invasion				0.049^*^			0.819
Negative	44	13 (29.5)	31 (70.5)		14 (31.8)	30 (68.2)	
Positive	34	3 (9.8)	31 (91.2)		10 (29.4)	24 (70.6)	
Perineural invasion				0.0001^*^			0.225
Negative	25	12 (48.0)	13 (52.0)		10 (40.0)	15 (60.0)	
Positive	53	4 (7.5)	49 (92.5)		14 (26.4)	39 (73.6)	

∆ χ^2^ test;

* Significant different, P<0.05;

† Mean age

### CXCL12 promoted PCa cell invasion and metastasis via CXCR4

**Fig. 3:**
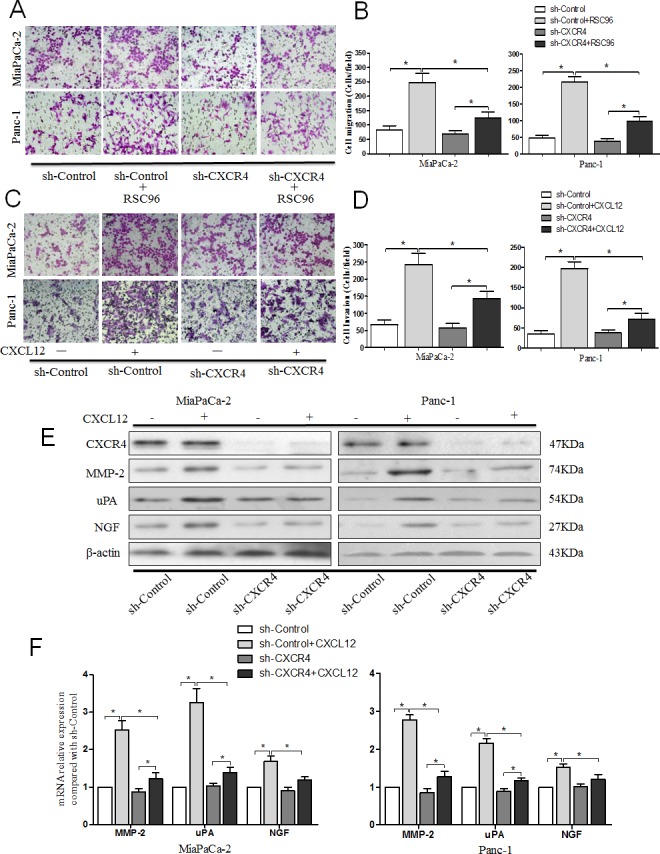
CXCL12 promoted pancreatic cancer cells metastasis and invasion via the receptor CXCR4 (A) The effects of the CXCL12/CXCR4 axis on PCa cell migration capability were assessed using a cell migration assay. The PCa cells were seeded into an uncoated chamber co-cultured with or without RSC96 for 24 h. (B) The number of migrated cells was quantified by counting the number of cells from 10 random fields at 200× magnification. (C) The effect of CXCL12/CXCR4 axis on PCa cell invasion capability was assessed using a Matrigel invasion assay. PCa cells were seeded into the Matrigel-coated invasion chamber pretreatment with or without CXCL12 for 48 h. (D) The number of invaded cells was quantified by counting the number of cells from 10 random fields at 200× magnification. The data are representative of 3 independent experiments. Column: mean (n=10); bar: SD; *P<0.05. (E) Western blot assays were performed to evaluate the effect of CXCL12/CXCR4 axis on the expression of CXCR4, MMP-2, uPA and NGF at protein level. (F) qRT-PCR was performed to evaluate the effect of CXCL12/CXCR4 axis on the expression of MMP-2, uPA and NGF at mRNA level. Column: mean; bar: SD; *P< 0.05.

To determine the effects of CXCL12/CXCR4 signaling on cell migration, PCa cells transfected with or without CXCR4 shRNA were indirectly co-cultured with RSC 96 cells. The number of migrating cells (MiaPaCa-2-shControl and Panc-1-shControl) is significantly increased in the co-cultured group compared with the single cultured group, while the migration capacity is dramatically inhibited with the CXCR4 shRNA (Fig. 3A and B). The results reveal that the medium in the lower chamber may contain some pro-migratory factors produced by RSC 96 cells for PCa cells and that the increased migration is dependent on the receptor CXCR4.

To determine the role of the CXCL12/CXCR4 axis in the enhanced invasive capacity of PCa cells, cells were cultured for 48 h with or without CXCL12. The results showed that the invasion ability (Fig. 3C and D) and the expression levels of the invasion-related genes (MMP-2 and uPA) of MiaPaCa-2-shControl and Panc-1-shControl cells are significantly increased after treatment with 100 ng/ml CXCL12 (Fig. 3E and F, P<0.05). The enhancement of tumor cell invasion is effectively countered by CXCR4 shRNA. In addition, as a control, we used BxPc-3 cells, which express low levels of CXCR4, stably transduced with CXCR4 (BxPc-3–CXCR4) or vector control (BxPc-3-control). We found that BxPc-3–CXCR4 cells after CXCL12 treatment acquire a stronger invasion capacity compared with BxPc-3-control cells. The expression levels of MMP-2, uPA and NGF are significantly increased as well (Supplementary Fig. 2). These data indicate that the activation of CXCL12/CXCR4 signaling enhances the invasive ability of PCa cells through up-regulating the expression of invasion-related genes (MMP-2 and uPA).

### Expression of CXCR4 and CXCL12 in RSC96 cells and DRGs

**Fig. 4:**
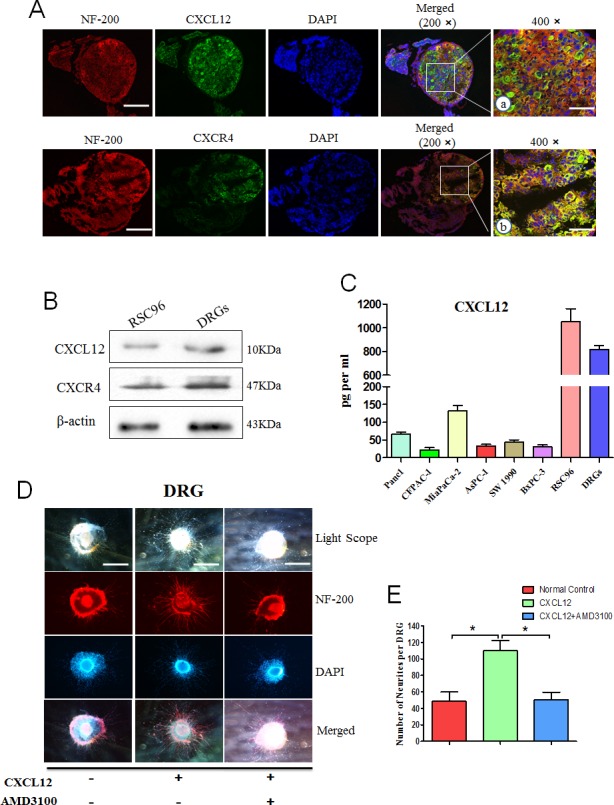
CXCL12/CXCR4 signaling pathway mediates the development of DRG The expression of CXCR4 and CXCL12 in DRGs and RSC96 cells was detected. (A) DRGs were labeled with fluorescence-conjugated NF-200 (red) with CXCL12 or CXCR4 specific antibodies. Nuclei were stained with DAPI (blue) (200× magnification, scale bar, 200μm). a and b represent 400× magnification pictures in the white border (scale bar, 100 μm); (B) The protein levels of CXCL12 and CXCR4 in DRGs and RSC96 cells were detected by western blot assays; (C) ELISA was performed to detect the expression of CXCL12 in conditioned media from pancreatic cancer cells, DRGs and RSC96 cells grown under serum-free condition for 72 h using a commercial kit. New-born rat DRGs were isolated and cultured in the medium containing CXCL12 (100 ng/ml) or both CXCL12 and the CXCR4 antagonist AMD3100 (2 μg/ml). (D) Five days after implantation, the DRGs were labeled with a fluorescence-conjugated NF-200 (red). Nuclei were stained with DAPI (blue). The cells were observed by an inverted light microscope imaging system 100× magnification (*P<0.05, scale bar, 500μm). (E) The number of outgrowth neurites was quantified (100× magnification). Column: mean; bar: SD; *P< 0.05.

DRGs were identified by immunofluorescence using anti-S100 (nerve tissue protein) and anti-NF200 (neurofilament protein) antibodies (Supplementary Fig. 1C). Double staining was performed for NF-200 with CXCR4 or CXCL12. As shown in Fig. 4A and B, both CXCR4 and CXCL12 are detected in RSC96 cells and DRGs. The cell culture supernatant was examined by ELISA. The results confirmed that CXCL12 secretion is high in the RSC96 Schwann cells and in DRGs, but the expression in PCa cells is low (Fig. 4C).

### CXCL12 accelerates the neurites outgrowth of DRGs

To explore the role of the CXCL12/CXCR4 axis in nerve growth, newborn rat DRGs were implanted in Matrigel with medium containing 100 ng/ml CXCL12. We observed neurite outgrowth from the DRGs after 24 h. The neurites with the exogenous CXCL12 group grew more luxuriant (Fig. 4D), and the number and length of neurite outgrowths are significantly increased compared with those in the control group (Fig. 4E, P<0.05). These effects could be inhibited by pretreatment with 2 μg/ml of AMD3100 (P<0.05). These results suggest that CXCL12 has a stimulating effect on the regeneration of DRG neurites.

### CXCL12/CXCR4 axis enhances the interaction between PCa cells and DRGs

To demonstrate that the CXCL12/CXCR4 axis is closely related to the PNI of PCa cells, an *in vitro* neural invasion model was constructed using MiaPaCa-2 cells co-cultured with newborn rat DRGs in Matrigel.

**Fig. 5:**
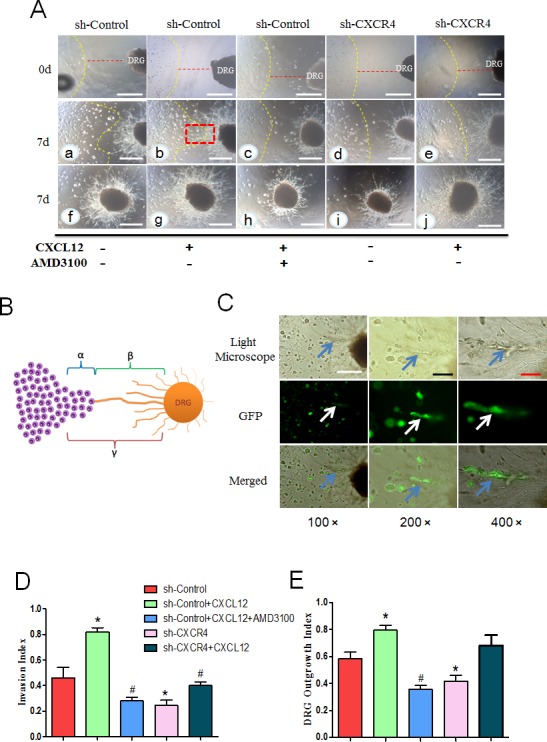
CXCL12/CXCR4 axis is involved in the interaction between pancreatic cancer cells and DRG (A) In the co-culture model, PCa cell clusters gradually migrated to the DRG, and neurite outgrowths extended from the DRG to the clusters, providing an invasive pathway for the clusters (a). This trend was significantly enhanced by adding CXCL12 (100 ng/mL) (b). AMD3100 impaired the promoting effect of CXCL12 on cancer cell cluster migration and neurite outgrowth (c). sh-CXCR4 blocked the effect of neurite-produced (d) or the exogenous CXC12 (e). Panels f, g, h, i and j display the entire shape of the DRG in each group after 7 days culture (100× magnification). The yellow line represents the initial frontier of the PCa cell colony at day 0. Scale bar, 500μm. (B) An illustration showing the calculation of nerve invasion index (α/γ) and DRG outgrowth index (β/γ). (C) The PCa cells infiltrated and migrated along the neurite. Different magnification (100×, 200×, and 400×) of the invaded cells in Fig. 5A (b) with a red dotted line was observed by fluorescent microscope under the light scope (blue arrow) and PCa cells transduced by green fluorescent protein vector (white arrow). Scale bar, 500μm; Black bar, 200μm; Red bar, 100 μm. (D) Invasion index (E) DRG outgrowth index, in the CXCL12 group were significantly increased compared with the control group. This increase was inhibited by pretreatment with the CXCR4 blocker AMD3100 and sh-CXCR4 (*P<0.05 vs. sh-Control, ^#^P<0.05 vs. sh-Control with CXCL12).

Seven days after implantation, we found that the PCa cells had invaded to the DRG neurites. The cancer cells facing the DRG formed peak-like clusters and gradually migrated to the DRG. Simultaneously, the outgrowth neurites facing the cancer cells extended directly toward the cancer cell clusters. This phenomenon is more obvious in the groups with the addition of exogenous CXCL12, where we observed a retrograde migration of the cancer cells along the contacting neurites after the neurite-cancer cell contact (Fig. 5A). Invasion index and DRG outgrowth index is also obviously higher than the control group (Fig. 5D and E). To further investigate the role of the CXCL12/CXCR4 axis in the interaction between PCa cells and DRGs, sh-CXCR4 cells (to block the expression of CXCR4 in cancer cells only) and AMD3100 (to block CXCR4 in both cancer cells and neurites) were used in the co-culture system. The results demonstrated that the migration ability and the mutual growth trend between the PCa cells and neurites in the sh-CXCR4 group are significantly suppressed compared with that in the control group. The AMD3100 pretreatment significantly inhibited PCa cells proliferation and migration, as well as DRG growth. After the treatment with CXCL12 in the sh-CXCR4 group, DRG growth is not significantly different, but the migration ability of the PCa cells and the reciprocal effect between the PCa cells and neurites are notably inhibited compared with that in the sh-Control group with CXCL12 (Fig. 5A, D and E).

In addition, we found that the NGF expression level of PCa cells is notably increased in response to the CXCL12 treatment (P < 0.05). After using CXCR4 shRNA to block the pathway, the increased expression is countered (Fig. 3E and F). Taken together, these findings indicate that CXCR4 blockade could attenuate CXCL12-induced PCa cell migration, invasion, and neural invasion.

### Relationship between CXCL12/CXCR4 axis and PNI of PCa *in vivo*

**Fig. 6:**
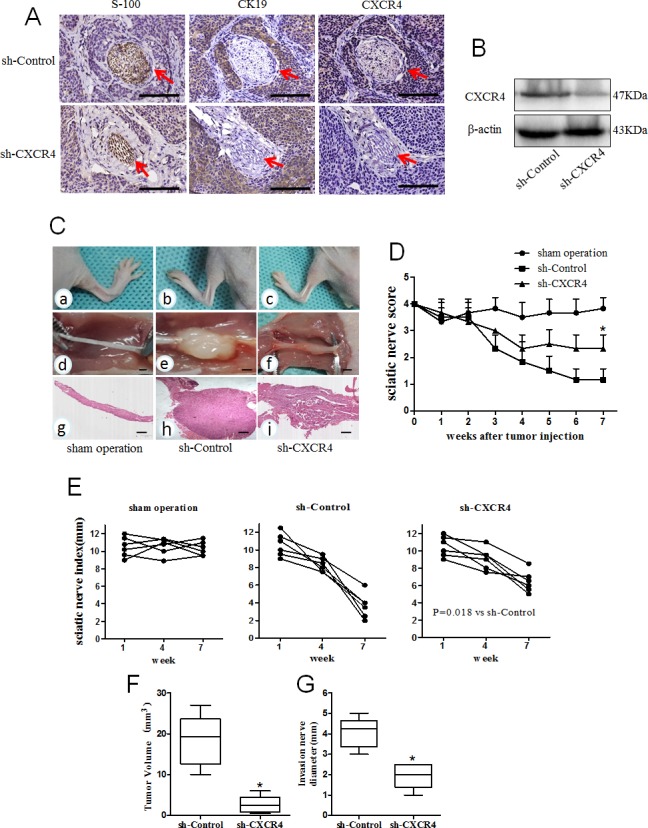
Relationship between CXCL12/CXCR4 axis and PNI of pancreatic cancer *in vivo* (A) Microscopic findings in mouse PNI models. Immunohistochemical staining of S100 (nerve tissue marker), CK19 (cancer cell marker) and CXCR4 were used to evaluate PNI *in vivo* neural invasion model. The tumor cells of sh-Control were arranged around nerves (the nerve indicated by red arrowheads). In contrast, sh-CXCR4 derived tumors exhibited no interaction between the tumor cells and nerves (Scale bar, 1mm); (B) Western blot of the resected tumors in mouse PNI models revealed a stable CXCR4 gene knockdown effect in sh-CXCR4 compared with sh-Control. MiaPaCa-2-shControl (n=6) and MiaPaCa-2-shCXCR4 (n=6) cells were implanted in a distal part of the left sciatic nerve, the right sciatic nerve served as a sham operation control (injected with saline, n=6). (C) Representative images of the spread length (in mm) between the first and fifth toes of the mouse hind limbs (a-c), xenograft tumor in situ of the sciatic nerve (d-f) and corresponding HE staining (g-i, 40× magnification) in different treatment groups (Scale bar, 1mm). (D) The mean left sciatic nerve scores in different groups were measured weekly for 7 weeks (*P< 0.05 vs. sh-Control). The sciatic nerve indexes (paw span in mm) (E) in sham operation group; (F) MiaPaCa-2-shControl group and (G) MiaPaCa-2-shCXCR4 group (P=0.018 vs. sh-Control) were measured at 1, 4, and 7 weeks after injection. The tumor volume (H) and invasion nerve diameter (I) were measured in sh-Control and the sh-CXCR4 groups (P values were determined by ANOVA, *P<0.05 vs. sh-Control).

To investigate these interesting findings *in vitro*, a neural invasion model with nude mice was used. A total of 24 mice were randomized into two groups: MiaPaCa-2-shControl and MiaPaCa-2-shCXCR4. The cell suspensions were injected s.c. on the midline of the mouse back. Eight weeks after injection, the tumors were resected for histologic examination to determine the PNI to nerves. The proteins were extracted and used for western blot assays to evaluate the correlation between CXCR4 and PNI. The results showed that PNI incidence in the sh-CXCR4 group is 33.3% (4/12, P = 0.011) which is significantly lower than that in the sh-Control group [83.3% (10/12)]. A significant decrease in the CXCR4 protein levels of the s.c. tumors derived from mouse perineural invasion models in sh-CXCR4 is shown compared with the sh-Controls (Fig. 6A and B).

Another *in vivo* model was performed to explore the effect of CXCR4-silencing in cancer cells on their invasion along nerves. The mice were randomized into three groups: sham operation; MiaPaCa-2-shControl and MiaPaCa-2-shCXCR4. The mice in the sh-CXCR4 group began to experience hind-limb dysfunction 10-15 days after cancer cell injection and developed left hind limb paralysis in the 6^th^ week. In comparison in the sh-Control group, the mice experienced hind-limb dysfunction after 7 days and developed left hind limb paralysis in the 4^th^ week. Five of the six mice in the sh-Control group are fully paralyzed at week 7, while only 1 mouse is paralyzed in the sh-CXCR4 group. Hind-limb functions are normal for all mice in the sham operation group (Fig. 6C). The sciatic nerve scores among the three groups of mice are significantly different (P < 0.001, Fig. 6D). The sciatic nerve index (the hind limb paw span) was used as an additional measure of sciatic nerve function. In the sh-CXCR4 group, the sciatic nerve index of the left hind limb is dramatically increased compared with the sh-Control group (Fig. 6E, P= 0.018). The mice were sacrificed at week 7, and the sciatic nerves were excised for HE staining (Fig. 6C). The degree of nerve invasion was determined by measuring the tumor diameter at 5 mm from the implantation site toward the spinal cord. The HE staining showed that the sh-CXCR4 mice had significantly decreased sciatic nerve diameters compared with controls, both at the primary tumor site and at 5 mm proximally along the sciatic nerve. The tumor volume and invasion nerve diameter in the sh-CXCR4 group are prominently reduced compared with those in the sh-Control group (Fig. 6F and G, P<0.05). These data demonstrate that the abrogation of CXCL12-CXCR4 signaling could inhibit tumor growth and invasion of the sciatic nerve toward the spinal cord.

## DISCUSSION

In the current study, we demonstrated a critical role for CXCL12/CXCR4 signaling in PNI progression and pathogenesis. CXCR4 expression significantly correlated with PNI as well as other major invasive parameters, including lymph node metastasis, TNM stage and vascular invasion in PCa patients. CXCR4 is highly expressed in PCa tissues with PNI compared with the non-PNI PCa tissues. We found that nerve tissues in the PCa specimens with PNI highly express CXCL12, and the cancer cells that expressed the CXCR4 receptor are arranged around nerves. The activation of CXCL12/CXCR4 signaling enhances PCa cells migration and invasion through up-regulating the expression of MMP-2 and uPA. In addition, our results demonstrated that CXCL12 clearly increase NGF expression in PCa cells and promote neurite regeneration via the receptor CXCR4. More importantly, the neural invasion model *in vitro* and *in vivo* showed that the abrogation of CXCL12/CXCR4 axis could suppress the PCa cell chemotactic migration to nerves and the invasion of the sciatic nerve toward the spinal cord. Collectively, to our knowledge, our studies demonstrate for the first time, that the CXCL12/CXCR4 axis could regulate the reciprocal signaling interactions between the peripheral nerves and PCa cells, thereby critically contributing to the PNI pathogenesis of PCa.

PNI as a distinct route of metastasis is associated with a poor prognosis and is more frequent than suspected, likely because neoplastic cells disseminating along nerve fascicles are spared by resectable surgery and cause recurrence. A complex network of chemokines and chemokine receptors exist in the tumor microenvironment. The chemokine receptor CXCR4 is a representative target gene [24-27]. Our study is the first to evaluate the expression of CXCL12 and CXCR4 in PCa specimens accompanied by PNI. Intriguingly, we found that the PCa cell lines rarely expressed CXCL12, but the majority of cancer cells surrounding or infiltrating in nerves showed distinct immunostaining of CXCR4 and CXCL12. These results suggested that certain factors in the tumor microenvironment may induce the expression of CXCR4 and CXCL12. Previous studies have reported that hypoxic conditions within solid tumors may induce CXCR4 expression and SDF-1 expression via hypoxia inducible factor (HIF)–1α contributing to the adhesion, migration and homing of circulating CXCR4-positive progenitor cells to ischemic tissue. Moreover, VEGF produced by tumor cells may induce CXCR4 expression in the tumor cell itself and/or in tumor-associated endothelial cells that facilitate both angiogenesis and metastasis of the primary tumor [28, 29].

The CXCL12/CXCR4 pathway has various functions in the nervous system, ranging from the trafficking of stem cells during embryogenic development to the effects on neuronal excitability and synaptic transmission [22]. The chemokine CXCL12, which is expressed at high levels in astrocytes, regulates the migration as well as patterning of neurons and microglial cells via its receptor CXCR4 in glial cells and specific subsets of neurons [23, 30-32]. Our study showed that in the peripheral nervous system, both CXCL12 and CXCR4 are expressed in Schwann cells and the entire DRG. CXCL12 promotes the neurite outgrowth of DRGs, and the stimulating effect could be inhibited by AMD3100, a CXCR4 specific antagonist. These data suggest that the growth and development of DRG is closely related to the CXCL12/CXCR4 pathway.

The CXCL12/CXCR4 axis also plays an important role in the PCa progression through tumor cell migration and invasion [19]. CXCR4 expression was significantly higher in the pancreatic tumor tissues compared with the normal pancreas [33]. Recent evidence has shown that multiple factors could enhance CXCR4 expression, including HIF-1, VEGF, activation of (NF-κB) and hepatocyte growth factor (HGF) [14, 34-36]. Consistent with previous reports, we showed that CXCL12 enhanced PCa cell migration and invasion, and the expressions levels of invasion-related genes, MMP-2 and uPA, were significantly up-regulated. Collectively, peripheral nerve-derived CXCL12 binding to CXCR4 of PCa cells promoted PNI through up-regulating the expressions of MMP-2 and uPA.

The tumor microenvironment in PCa is characterized by a complex interplay between the normal host epithelial cells, invading tumor cells, stromal fibroblasts, inflammatory cells, proliferating endothelial cells, an altered ECM, and growth factors activating oncogenic signaling pathways by autocrine and paracrine mechanisms [37]. Peripheral nerves, as novel and important members of the tumor microenvironment, have recently gained attention as being important. PNI is no longer presumed to be simply the growth of tumor cells along a “path of low resistance” but is rather considered the result of an active and specific reciprocal interaction between peripheral nerves and malignant cells [3]. Our results demonstrated that migrating PCa cells were evidently increased in the group co-cultured with Schwann cells, and the chemotactic migration of PCa cells towards Schwann cells could be inhibited by the abrogation of CXCR4. In the model of PNI *in vitro*, CXCL12 promoted the directional outgrowth of DRG projecting toward PCa cells and these, in turn, experienced early morphologic changes at the migration front, eventually leading to clusters of malignant cells around the neurites establishing PNI. The neural tropism of the PCa cells was significantly decreased by blocking the CXCL12/CXCR4 pathway. These findings indicate a mutual tropism and paracrine interaction between neurons, Schwann cells and cancer cells: nerves provide a prosperous environment for tumor growth via secreting CXCL12, and the interaction has beneficial effects on the growth of both the nerves and the tumor. Taken together, CXCL12 derived from peripheral nerves enhanced the invasion and metastasis of the CXCR4-positive PCa cells. In addition, the optimum dose gradient of CXCL12 induced chemotactic migration of PCa cells toward the nerves and, ultimately, metastatic spread along the alternative route.

Neurotrophic factors and, more recently, chemokines have been identified as molecular determinants of PNI [4, 38-40]. We clearly observed that although the interaction between the PCa cells and neurites was significantly decreased after blocking the CXCL12/CXCR4 axis, there was still mutual taxis movement between them. Cancer cells do not or rarely express CXCL12, and should have no effect on DRGs. Moreover, the nerve cells (DRG) are the only source of CXCL12. Hence, the interaction is more likely to be unidirectional rather than bidirectional. Therefore, we investigated NGF expression in PCa cells after CXCL12 treatment. The results showed that CXCL12 increased NGF expression levels, and the effect was counterbalanced by sh-CXCR4. These data indicate that the CXCL12/CXCR4 axis can promote neural tropism by up-regulating NGF expression.

Furthermore, recent research shows another seven-transmembrane receptor CXCR7 as a second receptor of CXCL12 [41]. It has been proposed that CXCR7 functions as a “decoy” receptor to generate a gradient of CXCL12 through ligand sequestration; the CXCL12 gradient would impact CXCR4 signaling and the chemoattraction of positive cells with CXCR4 through controlling ligand availability [42]. However, unlike CXCR4/G protein-mediated signaling, CXCL12 binding to CXCR7 may only signal through β-arrestin, the activation of which leads to stimulation of the MAP kinase cascade [43, 44]. CXCR7 acts as a sink for CXCL12 and controls cell migration by ligand sequestration [45]. An in-depth research for the role of CXCR7, especially regarding the mechanisms that alter overall availability, distribution and gradients of CXCL12 in tumor microenvironments to regulate the invasion and metastasis of CXCR4 expressing cells, is imperative in the future.

AMD3100 (Mozobil, plerixafor), CXCR4 inhibitor, has been approved by the Food and Drug Administration (FDA) as a mobiliser of hematopoietic CD34+ cells from the bone marrow to the circulation. Several clinical trials performed to date in acute myelocytic leukemia (AML) and chronic lymphocytic leukemia (CLL) demonstrated that combined therapy of plerixafor with conventional chemotherapy is safe and does not affect hematological recovery. However, the benefit of combination treatment still needs to be proven in further clinical trials. Another clinical trials indicated that CTCE-9908, an inhibitor of the CXCR4/CXCL12 pathway, reduced refractory metastatic or locally advanced solid tumors with bevacizumab in several solid tumors [46, 47]. Our study confirmed that the CXCR4/CXCL12 axis correlated with PCa cell invasion and metastasis and played a pivotal role in PNI, the distinct metastatic route. It is imperative to test the effects of the CXCR4/CXCL12 pathway inhibitor on PCa, and we believe that it will provide encouraging results.

In conclusion, our results indicate a mutual tropism and paracrine interaction between nerves and cancer cells: peripheral nerve-derived CXCL12 stimulates the proliferation and invasion of CXCR4-positive tumor cells. Moreover, CXCR4-expressing tumor cells migrate along the CXCL12 gradient derived from nerves, eventually leading to metastatic spread along the alternative route establishing PNI. Our study not only indicates a pathogenesis for PCa PNI, but also provides evidence that CXCL12/CXCR4 axis might be a novel therapeutic target to prevent tumor perineural dissemination in PCa.

## MATERIALS AND METHODS

### Cell lines, culture conditions and reagents

The human PCa cell lines CFPAC-1, BxPC-3, MiaPaCa-2, AsPC-1, SW1990, Panc-1, and the rat Schwann cells RSC96 were purchased from the Chinese Academy of Sciences Cell Bank of Type Culture Collection (CBTCCCAS). According to the instructions, all cell lines were cultured in the proper medium (HyClone, Logan, USA) supplemented with 10% fetal bovine serum (FBS), 100 μg/ml ampicillin and 100 μg/ml streptomycin. The cultures were incubated at 37°C in a humidified atmosphere containing 5% CO_2_.

Recombinant human CXCL12 was purchased from PeproTech (Rocky Hill, USA). The pharmacological reagent AMD3100 was purchased from Sigma (St. Louis, MO, USA). Antibodies were purchased from the following sources: anti-CXCR4, anti-CXCL12, anti-NGF, anti-β-actin (Santa Cruz Biotechnology, Santa Cruz, CA, USA), anti-MMP-2, anti-uPA (Bioworld, Minneapolis, MN, USA), anti-S100 and anti-NF200 (Abcam, USA).

### Stable transfection of the CXCR4 shRNA vector and CXCR4 expression vector

In a previous study by our team, we screened and constructed stable CXCR4 shRNA expression and control vectors [19]. The shRNA against CXCR4 (5′-gatccTGAGAAGCATGACGGACAAt tcaagagaTTGTCCGTCATGCTTCTCAttttttggaaa-3′) and the negative control shRNA (5′-gatccTTCTCCGAACGTGTCACGTttca agagaACGTGACACGTTCGGAGA

Attttttggaaa-3′) were purchased from GenePharm (Shanghai, China). The stable CXCR4-suppressed PCa cells and the Control PCa cells were named sh-CXCR4 and sh-Control, respectively. The effect of gene silencing was evaluated by qRT-PCR and western blot (Supplementary Fig. 1A and B). Lentiviral vectors for CXCR4-GFP or GFP control were used to stably transduce BxPc-3 cells to create BxPc-3-CXCR4 or BxPc-3-control cells.

### Immunohistochemistry

A total of 78 pancreatic carcinoma samples and 8 normal pancreatic tissues were obtained from the Department of Hepatobiliary Surgery, the First Affiliated Hospital of Xi'an Jiaotong University between 2010 and 2013. The pathological TNM status was assessed according to the criteria of the sixth edition of the TNM classification of the American Joint Commission on Cancer (2010) [48]. The definition of PNI was determined as described previously [9], and other pathologic factors were examined by two pathologists. The results are summarized in Table 1.

Immunohistochemical staining for S-100, CK19 (Maxim, Fuzhou, China), CXCR4 and CXCL12 were performed using the SABC kit (Maxim, Fuzhou, China) according to the manufacturer's instructions. Briefly, the tissue sections were incubated with primary antibodies for S-100 (1:100), CXCR4 (1:50) and CXCL12 (1:50) overnight at 4°C, incubated with the appropriate biotinylated secondary antibody for 30 min at room temperature, followed by 30 min of incubation with streptavidin peroxidase (Dako LSAB+HRP kit). After rinsing, the results were visualized using DAB, and the slides were counterstained with hematoxylin.

The study protocol and consent forms were approved by the relevant ethical committee of the First Affiliated Hospital of Medical College, Xi'an Jiaotong University, China.

### Immunofluorescence

For fluorescent immunocytochemistry, the pancreatic tumor cell lines and the neurite outgrowths from newborn rat DRGs (cultured after 5 days) were fixed for 20 min in 4% paraformaldehyde in PBS, and the endogenous peroxidase activity was quenched by 3% hydrogen peroxide. The specimens were permeabilized with 0.2% Triton X-100 containing 1% normal goat serum (NGS) in PBS for 20 min on ice, pre-blocked for 30 min with bovine serum albumin (BSA) at 37°C, and incubated with primary antibody overnight at 4°C. Staining was detected with fluorescein-conjugated secondary antibodies (Jackson Immuno Research).

DRGs were quickly removed from the newborn rat, frozen in dryice, and preserved at −80°C. Sections 6-μm thick were cut on a cryostat (Leica Inc.) and stored at −80°C until use. Sections were pre-blocked for 1 h with BSA at room temperature, and the NF-200 antibody was co-incubated with the CXCR4 or CXCL12 antibody overnight at 4°C. Staining was detected with its corresponding fluorescein-conjugated secondary antibodies (Jackson Immuno Research).

Slides were mounted and examined using a Nikon Instruments confocal microscope.

### Cell migration and invasion assay

The transwell chamber (pore size, 8.0 μm; Millipore, Billerica, USA) without (for migration assay) or with Matrigel (for invasion assay) coating was inserted into a 24-well culture plate. For the migration assay, the PCa cells (100 μl, 5×10^4^) suspended in DMEM containing 1% FBS were placed in the upper chamber with or without RSC96 cells (500 μl, 1×10^5^) in the lower chambers in 10% FBS-containing medium. The transwell chamber was incubated for 24 h.

For the invasion assay, 8 μm pore inserts were coated with Matrigel (BD Biosciences, Oxford, UK). PCa cells in log phase growth were cultured in 6-well plates in medium containing 1% FBS for 24 h before drug treatment. The PCa cells (100 μl, 5×10^4^) suspended in DMEM containing 1% FBS with or without 100 ng/ml CXCL12 were seeded in the top chamber, and 500 μl of medium containing 10% FBS was placed in the lower chamber as a chemoattractant. The transwell chamber was incubated for 48 h.

The migrated and invaded cells on the bottom surface of filter were fixed in methanol and stained with crystal violet (Boster Biological Technology Ltd., Wuhan, China). Cell migration and invasion was determined by counting the stained cells under a light microscope in 10 randomly selected fields.

### Western blot analysis

The DRGs and cells were lysed using cell lysis buffer with protease inhibitors (Roche, Penzberg, Germany). Equivalent amounts of proteins were electrophoretically resolved on a denaturing SDS polyacrylamide gel and electro-transferred onto nitrocellulose membranes. The membranes were initially blocked with 5% nonfat dry milk in Tris-buffered saline (TBS) for 2 h and then probed with antibodies against CXCL12, CXCR4, MMP-2, uPA, and NGF. After co-incubation with the primary antibodies at 4°C overnight, the membranes were hybridized with the appropriate goat anti-mouse or anti-rabbit secondary antibody (Sigma-Aldrich) for 1 h at room temperature. Equal protein sample loading was monitored using an anti-β-actin antibody. The probed proteins were detected by using enhanced chemiluminescence (Millipore).

### Real-time PCR assay

Total RNA was extracted using the Fastgen200 RNA isolation system (Fastgen, Shanghai, China) according to the manufacturer's protocol. Total RNA was reverse-transcribed into cDNA using the Prime Script RT reagent kit (TaKaRa, Dalian, China). Real-time PCR was used to quantitatively examine the expression of CXCR4, MMP-2, uPA and NGF at the mRNA level. Real-time PCR was conducted according to a previous report [20, 49]. The PCR primer sequences for CXCR4, CXCL12, MMP-2, NGF, uPA and β-actin are shown in Additional file 1: Table S1. The comparative C (T) method was used to quantitate the expression of each target gene using β-actin as the normalization control.

### Enzyme-linked immunosorbent assay (ELISA)

The cells were conditioned in serum-free medium for 72 h. The culture media were collected, centrifuged at 1500 r.p.m. for 5 min to remove particles, and supernatants were frozen at −80°C until use. The production of CXCL12 in the supernatants of PCa cells and RSC96 was assessed by ELISA using a commercially available ELISA kit (R&D Systems, USA) according to the manufacturer's recommendations.

### Perineural invasion model for assessment of nerve-cancer cell interactions

Newborn rats were purchased from the laboratory animal center of the Xi'an Jiaotong University, sacrificed with carbon dioxide and sterilized with 75% ethanol. DRGs from the lumbar areas were dissected, collected into medium (DMEM/F12), stripped of meninges and nerve stumps, and then implanted into a drop of growth factor – depleted liquid Matrigel (BD Biosciences, Oxford, UK). After solidification, medium (DMEM/F12 containing 10% FBS) was carefully added, and in accordance with routine culture, half of the medium was changed every 2 days. Seven days after implantation, the DRGs were identified by histology and immunofluorescence for S-100 and NF-200 (Supplementary Fig. 1C).

The PNI model was established to simulate the microenvironment of the tumor and peripheral nerves as described previously [24, 50-52]. DRGs from newborn rats were stored on ice in DMEM and seeded in 24-well Petri dishes containing 25 μl of Matrigel approximately 1 mm from a colony of carcinoma cells suspended in 25 μl of Matrigel under an anatomical microscope. To exclude the possibility of nonspecific migration of cancer cells, an additional 25 μl of Matrigel containing no DRG was positioned on the opposite side-rear side. The dishes were placed in an incubator at 37°C saturated with 5% CO_2_ in a humidified atmosphere for 20 min to allow for Matrigel polymerization. After solidification, the medium (DMEM/F12 containing CXCL12 and/or the CXCR4 antagonist AMD3100) was added and renewed by half every 2 days. Photographic documentation of the cell suspensions on the two adjacent sides was performed with an inverted light microscope imaging system (Ti-E; Nikon Instruments Inc, Shanghai, China) and a Nikon Instruments confocal microscope. To conduct quantitative analysis on the results of the co-culture model, we defined the minimum distance between the edge of the cancer cells and the edge of DRG as parameter γ, the migration distance of cancer cells towards DRG as parameter α, and the DRG outgrowth length towards cancer cells as parameter β. The invasion index=α/γ, and the DRG outgrowth index=β/γ (Fig. 4B). The migration distance was measured by the image analysis software of the microscope imaging system. (NIS-Elements, Nikon Instruments Inc, Shanghai, China)

### *In vivo* PNI model

The PNI model was performed as described previously [53]. The mice were anesthetized, and 7×10^6^ cells in 100 μl of cell suspension were injected sciatic nerve (s.c.) on the midline of their backs at two sites using an inoculator fitted with a 23-gauge needle. Eight weeks after injection, the tumor was resected. The samples were processed for histologic examination to examine PNI to nerves.

To evaluate the effect of CXCL12/CXCR4 axis on the cancer cells invasion along the nerves, the neural invasion model was performed essentially as described previously [54]. PCa cells were microscopically injected into the sciatic nerve of the anesthetized nude mice, distal to the bifurcation of the tibial and common peroneal nerves. Microinjection of 3 μl of cell suspension at a concentration of 1×10^5^ cells per microliter was performed using a 5 μl microsyringe. To evaluate the sciatic nerve function, which innervates the hind limb paw muscles, we measured the following parameters: (a) gross behavior, which was monitored for 10 min weekly for signs of repetitive biting of the hind limb; (b) limb function, which was graded according to the hind limb paw response to manual extension of the body from 4 (normal) to 1 (total paw paralysis); and (c) the sciatic nerve function index, which was calculated as the spread length (in mm) between the first and fifth toes of the mouse's hind limbs weekly for 7 weeks. A total of 3 μl of physiological saline was microinjected into the right sciatic nerve served as a sham operation control.

### Statistical analyses

All statistical analyses were performed using SPSS 13.0 software. All data are expressed as the means±standard deviation (SD). The significance of the patient specimen data was determined using the Pearson correlation coefficient or Fisher's exact test. Differences between the groups were determined by analysis of variance (ANOVA), followed by the Bonferroni's correction for multiple comparisons. *P*< 0.05 was considered significant. All experiments were repeated independently at least three times.

## SUPPLEMENTARY MATERIAL TABLE AND FIGURES



## Abbreviations

DRG: dorsal root ganglia
PCa: pancreatic cancer
PNI: perineural invasion
NGF: nerve growth factor
MMPs: matrix metalloproteinases
uPA: urokinase-type plasminogen activator
