# Supplementary material for: Stromal-derived factor-1α/CXCL12-CXCR4 chemotactic pathway promotes perineural invasion in pancreatic cancer
Source: Oncotarget. 2014 Dec 26;6(7):4717–32. doi: 10.18632/oncotarget.3069 (PMC4467110; doi:10.18632/oncotarget.3069)
Supplement: Supplementary file 1 [file oncotarget-06-4717-s001.pdf]

# Stromal-derived factor-1α/CXCL12-CXCR4 chemotactic pathway promotes perineural invasion in pancreatic cancer

**Supplementary Material**

**Table S1: PCR Primer Sequences**

| Genes | Primer Sequence |
|---|---|
| Human | |
| β-actin | P1: 5'- AGCGAGCATCCCCCAAAGTT -3' |
| | P2: 5'- GGGCACGAAGGCTCATCATT -3' |
| CXCL12 | P1: 5'- GTGGTCGTGCTGGTCCTC -3' |
| | P2: 5'- CACACTTGTCTGTTGTTGTTCTTC -3' |
| CXCR4 | P1: 5'- TCTGTGACCGCTTCTACC -3' |
| | P2: 5'- AGGATGAGGATGACTGTGG -3' |
| NGF | P1: 5'- GTCATCATCCCATCCCATCTTCC -3' |
| | P2: 5'- CACTGTTGTTAATGTTCACCTCTCC -3' |
| uPA | P1: 5'- TAAGAGCTGGTGTCTGATTG -3' |
| | P2: 5'- TTGGATGAACTAGGCTAAAA -3' |
| MMP-2 | P1: 5'- GATGATGCCTTTGCTCGTGC -3' |
| | P2: 5'- CAAAGGGGTATCCATCGCCA -3' |
| Rat | |
| β-actin | P1: 5'- TCAGGTCATCACTATCGGCAAT -3' |
| | P2: 5'- AAAGAAAGGGTGTAAAACGC -3' |
| CXCL12 | P1: 5'- ATGTCGCCAGAGCCAACG -3' |
| | P2: 5'- TCCACTTTAATTTCGGGTCAATGC -3' |
| CXCR4 | P1: 5'- CCGTGGCTGACCTCCTCTTTG -3' |
| | P2: 5'- TGGTGGCGTGGACAATGGC -3' |

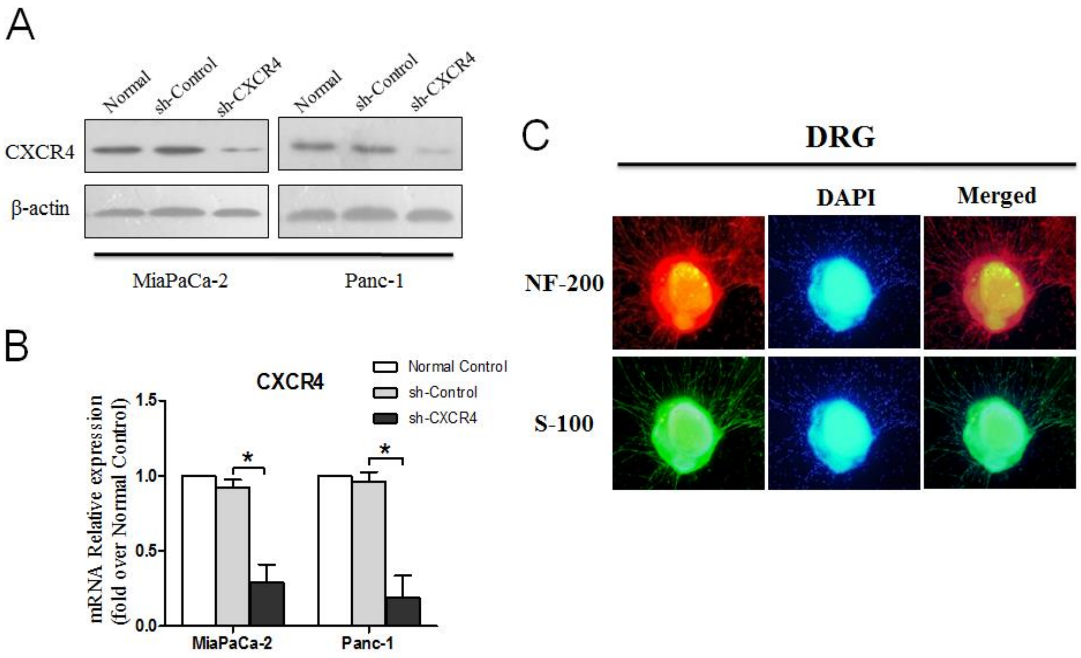


**Supplementary Fig. 1:** The establishment of PCa cells with stable CXCR4-silencing. After transfection with control shRNA or CXCR4 shRNA, the expression level of the CXCR4 protein (A) and mRNA (B) in Panc-1 and MiaPaCa-2 cells was evaluated by western blot and real-time PCR. The expression level of each target gene was quantified relative to β-actin as a normalization control. (C) DRGs were labeled with fluorescence-conjugated S-100 (green) and NF-200 (red) specific antibodies. Nuclei were stained with DAPI (blue; 100× magnification);

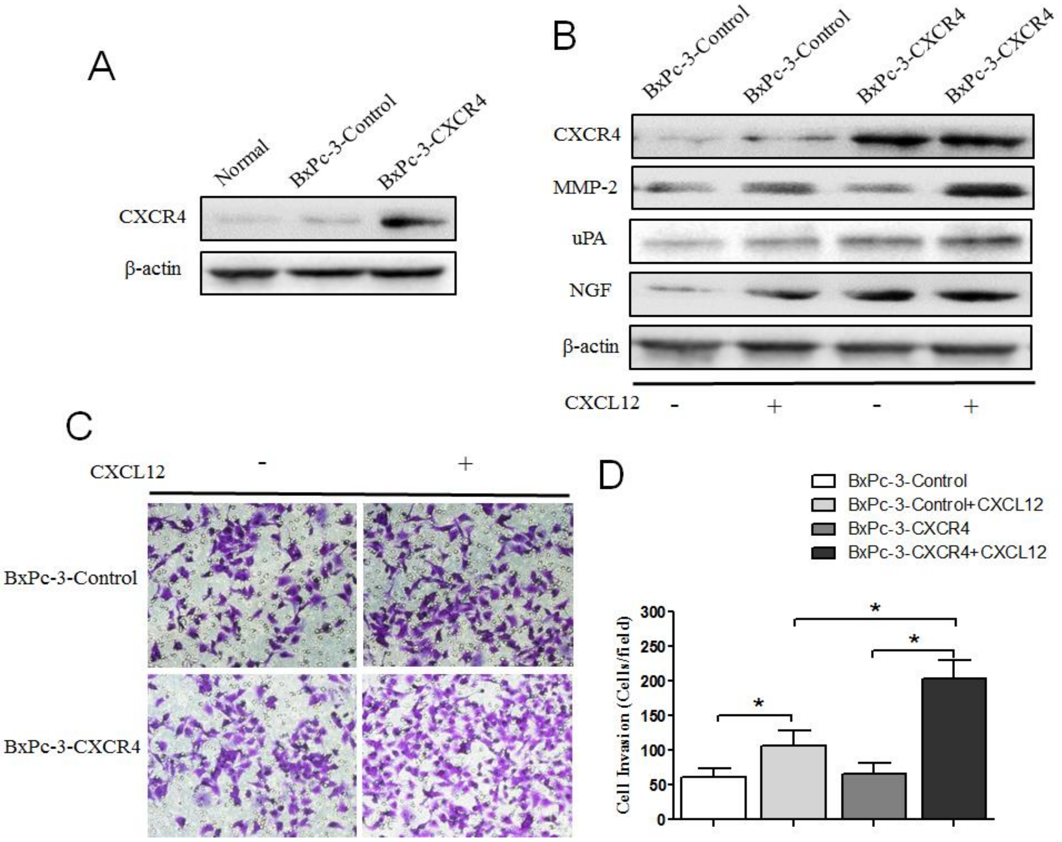


**Supplementary Fig. 2:** (A) The expression level of CXCR4 protein in BxPc-3 cells transduced by lentiviral vectors for CXCR4-GFP or GFP control was evaluated by western blot. (B) Western blot was performed to evaluate the effect of enforced expression of CXCR4 in BxPc-3 cells on the MMP-2, uPA and NGF at protein level. (C) A Matrigel invasion assay was performed to evaluate the invasion capacity of BxPc-3-CXCR4 cells. (D) The number of invaded cells was quantified by counting the number of cells from 10 random fields at 200× magnification. The data are representative of 3 independent experiments. Column: mean (n = 10); bar: SD; *$P<$ 0.05.
